# Gram positive and Gram negative bacteria differ in their sensitivity to cold plasma

**DOI:** 10.1038/srep38610

**Published:** 2016-12-09

**Authors:** Anne Mai-Prochnow, Maryse Clauson, Jungmi Hong, Anthony B. Murphy

**Affiliations:** 1 CSIRO Manufacturing, PO Box 218, Lindfield NSW 2070 Australia; 2 National Engineering School of Agronomy and Food Science, Nancy, France; 3 School of Physics, University of Melbourne VIC 3010, Australia

## Abstract

Cold atmospheric-pressure plasma (CAP) is a relatively new method being investigated for antimicrobial activity. However, the exact mode of action is still being explored. Here we report that CAP efficacy is directly correlated to bacterial cell wall thickness in several species. Biofilms of Gram positive *Bacillus subtilis*, possessing a 55.4 nm cell wall, showed the highest resistance to CAP, with less than one log_10_ reduction after 10 min treatment. In contrast, biofilms of Gram negative *Pseudomonas aeruginosa*, possessing only a 2.4 nm cell wall, were almost completely eradicated using the same treatment conditions. Planktonic cultures of Gram negative *Pseudomonas libanensis* also had a higher log_10_ reduction than Gram positive *Staphylococcus epidermidis.* Mixed species biofilms of *P. aeruginosa* and *S. epidermidis* showed a similar trend of Gram positive bacteria being more resistant to CAP treatment. However, when grown in co-culture, Gram negative *P. aeruginosa* was more resistant to CAP overall than as a mono-species biofilm. Emission spectra indicated OH and O, capable of structural cell wall bond breakage, were present in the plasma. This study indicates that cell wall thickness correlates with CAP inactivation times of bacteria, but cell membranes and biofilm matrix are also likely to play a role.

Plasma is ionized gas and can be generated using a range of gases, including argon, helium, nitrogen and compressed air. Plasma contains radicals, excited molecules, charged particles and UV photons. Cold atmospheric plasma (CAP) is active towards a broad spectrum of microorganisms. There is an active debate about which plasma species are responsible for microbial inactivation, with reactive oxygen, hydrogen peroxide (H_2_O_2_) and UV photons the most likely candidates^1^,^2^,^3^,^4^. Many studies have tested the antibacterial activity of CAP *in vitro*, but only very limited data on clinical trials have been reported to date^5^,^6^. It appears that the antimicrobial efficiency of CAP depends on specific properties of the devices used, making it challenging to investigate the mode of action.

The commercially-available CAP-producing device kINPen med was specifically designed for biomedical applications. The device has the same basic plasma properties as the widely-used standard plasma source “kINPen 09” but special attention has been paid to safe and easy operation in medical settings^7^,^8^,^9^. It has a DC power supply and can be used with a range of rare gases. A number of studies have shown its antibacterial effectiveness^10^,^11^.

Cold plasma is hypothesised to have different targets within the cell, including cell membrane and cell wall, DNA and intracellular proteins^4^,^12^. Plasma species were shown to be able to break important bonds in the cell wall (peptidoglycan) structure in Gram positive bacteria^13^,^14^ as well as leading to membrane lipid peroxidation in Gram negative bacteria^15^. This disruption of the outer shell of the cell will lead to leakage of cellular components, including potassium, nucleic acid and proteins. After the cell wall is broken, reactive species can penetrate into the interior of the cell and further damage DNA and intracellular protein from oxidative or nitrosative species^12^.

In bacteria, the Gram stain provides an important classification system, as several cell properties can be correlated with the cell envelope. Gram positive bacteria possess a thick (20–80 nm) cell wall as outer shell of the cell. In contrast Gram negative bacteria have a relatively thin (<10 nm) layer of cell wall, but harbour an additional outer membrane with several pores and appendices. These differences in the cell envelope confer different properties to the cell, in particular responses to external stresses, including heat, UV radiation and antibiotics.

Most *in vitro* studies focus on investigating plasma mediated killing of laboratory-cultured single-species planktonic cells. However, this does not resemble the natural conditions of bacterial existence. The vast majority of bacteria live in aggregates attached to a surface in often multi-species biofilms. Bacterial biofilms cause problems in several industries by colonizing factory equipment and contaminating products. They are also a major contributor to human infections and are particularly hard to eradicate with antibiotic therapy. Biofilms promote bacterial survival in the environment, as they coordinate group behaviour, enhance metabolic interactions, enhance gene transfer, produce a protective exopolysaccharide matrix from the cells and increase antibiotic resistance^16^,^17^,^18^,^19^,^20^.

Here we show that cold-plasma-induced bacterial biofilm killing is correlated with the thickness of the bacterial cell wall, but additional factors are involved in determining sensitivity to CAP inactivation. Using a commercially-available plasma source, a much higher reduction in cell numbers is achieved for Gram negative bacteria than Gram positive bacteria, independent of planktonic or biofilm mode of growth. Moreover, clinically-relevant *P. aeruginosa* is found to have a higher resistance to plasma treatment when cultured with *S. epidermidis* in mixed species biofilms. This has implications for eradicating environmental biofilms and treating clinical significant infections, in which bacteria are known to often occur as multispecies communities.

## Results

### CAP inactivation of Gram positive and Gram negative bacteria in biofilms

**Figure 1:**
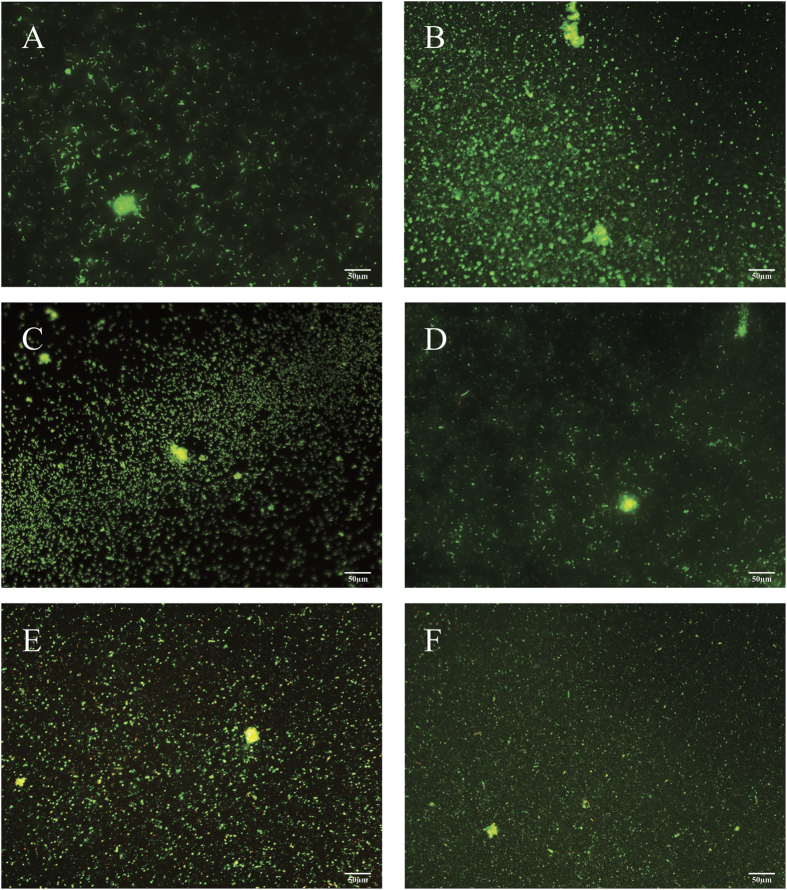
Images of Live/Dead stained biofilms. Biofilms were incubated on coupons for 24 hours before staining with Syto9 and propidium iodide. Images were taken with an Olympus IX83 fluorescence microscope using CellSense software. (**A**) *B. subtilis*, (**B**) *P. aeruginosa*, (**C**) *K. carniphila*, (**D**) *P. libanensis*, (**E**) *S. epidermidis*, (**F**) *E. cloacae*.

Biofilms of six different species (3 Gram positive and 3 Gram negative) were allowed to form on stainless steel coupons for 24 h. Biofilm formation was observed to be similar for all species after 24 hours (Fig. 1). Strains had single attached cells and small microcolonies were beginning to form. Only isolated dead (red) cells were observed with the majority being green (viable) cells.

**Figure 2:**
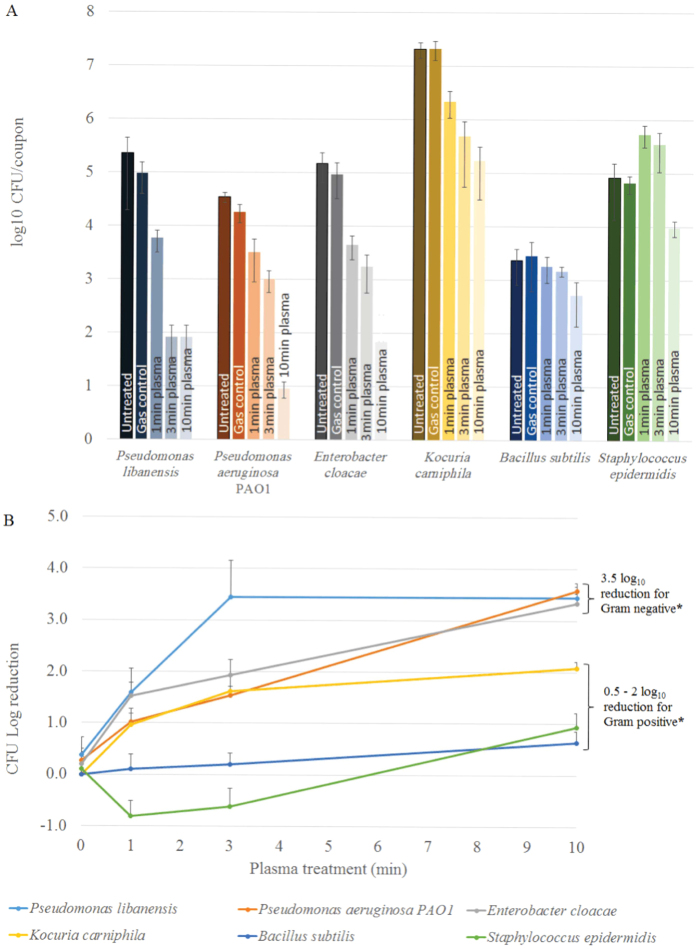
CAP treatment of single-species bacterial biofilms. Biofilms were grown on stainless steel coupons for 24 h before plasma treatment for 1, 3 or 10 min using the kINPen med with argon feeding gas at 3.1 slm. After treatment, cells were scraped from the coupon and dilutions plated onto nutrient agar (**A**) CFU numbers of untreated, gas control, 1, 3 and 10 min CAP, respectively. (**B**) CFU log_10_ reductions after 0 (gas control), 1, 3 and 10 min plasma treatment. The error bars represent standard deviations for 3 biofilm samples. *The CFU log_10_ reduction values for Gram negative bacteria at 10 min treatment show a statistically significant difference from the log_10_ reduction values of Gram positive bacteria at 10 min treatment (p < 0.05).

All biofilms were tested for inactivation rates using the kINPen med operated with argon gas (Fig. 2). The three Gram negative species showed a higher log_10_ reduction in colony forming units (CFU) than the three Gram positive strains after 10 min CAP treatment compared to the untreated controls. *B. subtilis* had the lowest CFU log_10_ reduction of only 0.6 and *Staphylococcus epidermidis* only had a 1 log_10_ reduction in CFU after ten minute CAP treatment. Another Gram positive species *Kocuria carniphila* reached a 2 log_10_ reduction in CFU after the 10 min CAP treatment. In contrast, biofilms of Gram negative species had a much higher log_10_ reduction after 10 min treatment. A similarly high reduction was observed for *P. aeruginosa, P. libanensis* and *E. cloaceae* biofilms ranging from 3.3 to 3.6 log_10_ reduction in CFU compared to the control (Fig. 2B). *P. libanensis* biofilms showed a rapid initial reduction in CFU, followed by a plateau. *E. cloacae, P. aeruginosa* and *K. carniphila* all showed a sharp decline in CFU in the first minute of CAP treatment, followed by a slower inactivation rate. For *B. subtilis* the inactivation occurred gradually over the 10 min, while *S. epidermidis* had no reduction in CFU at the 1 and 3 min time-point and inactivation only occurred after 10 min CAP treatment.

A Student’s *t*-test confirmed that the difference in log_10_ reduction comparing the Gram positive to the Gram negative strains is significant (p = 0.034) for the 10 min time-point. At 3 min and at 1 min plasma treatment the p-values were 0.095 and 0.058, respectively, indicating that the difference between the two groups of bacteria is not significant.

### Comparing CAP induced CFU reduction of bacteria in biofilms with cell wall thickness

**Figure 3:**
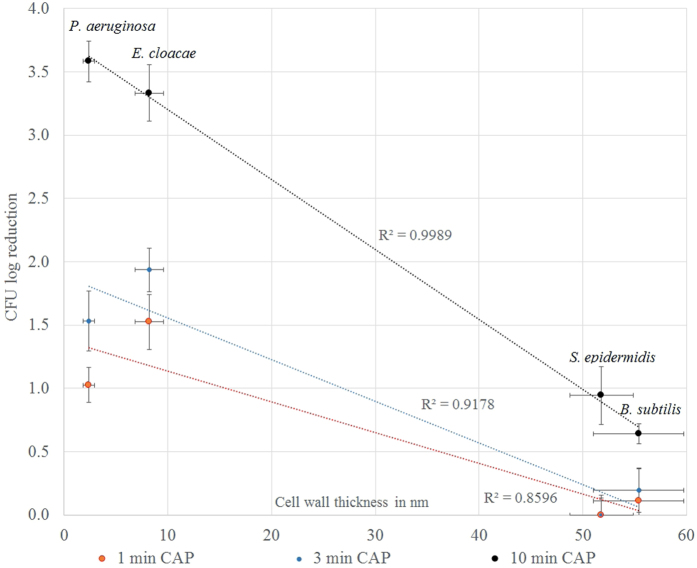
Variation of CFU log10 reduction at 1, 3 and 10 min plasma treatment of Gram positive (*S. epidermidis, B. subtilis*) and Gram negative (*P. aeruginosa, E. cloacae*) bacteria in biofilms with cell wall thickness.

A profound difference of CFU log_10_ reductions after CAP treatment was observed between Gram positive and Gram negative species biofilms, prompting us to investigate a possible correlation of CAP inactivation rates with cell wall dimensions. Values of cell wall thickness were sourced from previous studies. For *P. aeruginosa, S. epidermidis* and *B. subtilis*, cell wall thickness had been determined previously as 2.41 ± 0.54 nm^21^, 51.8 ± 3.1 nm^22^ and 55.4 ± 4.4 nm^23^,^24^,^25^, respectively. The cell wall thickness of *E. cloacae* was measured at approximately 8.2 ± 1.4 nm from high resolution images from previous studies^26^,^27^. No indication of cell wall thickness measurements or high resolution TEM images were found for *P. libanensis* and *K. carniphila* and these organisms were therefore not included in the analysis. A correlation between number of cells killed (CFU log_10_ reduction) after 1, 3 and 10 min of CAP treatment and cell wall thickness is shown in (Fig. 3).

There is a good correlation of CFU log_10_ reduction with cell wall thickness for all time-points (R^2^ = 0.86; 0.92 and 0.99 for 1, 3 and 10 min CAP, respectively). For 10 min CAP treatment, small differences in cell wall thickness within the group of Gram negative and Gram positive leads to a difference in CAP sensitivity. For 1 min and 3 min CAP treatment the correlation within Gram negative and the Gram positive group is reversed, i.e. *E. cloacae* with a thicker cell wall than *P. aeruginosa* had a higher log_10_ reduction than *P. aeruginosa.*

### CAP treatment of mixed-species biofilms

**Figure 4:**
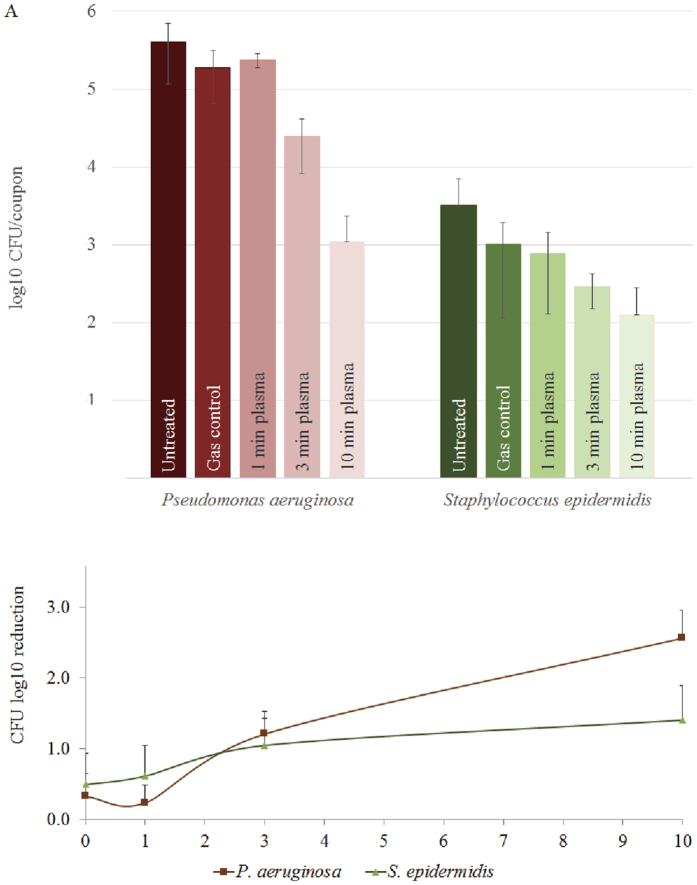
CAP treatment of mixed-species bacterial biofilms. Mixed biofilms of *P. aeruginosa* and *S. epidermidis* were grown on stainless steel coupons for 24 h before plasma treatment for 1, 3 or 10 min using the kINPen med with argon feeding gas at 3.1 slm. After treatment, cells were scraped from the coupon and dilutions plated onto Pseudomonas agar (*P. libanensis*) and Mannitol salt agar (*S. epidermidis*). (**A**) CFU numbers of untreated, gas control, 1, 3 and 10 min CAP, respectively. (**B**) CFU log_10_ reductions after 0 (gas control), 1, 3 and 10 min plasma treatment. The error bars represent standard deviations for 3 biofilm samples.

In natural habitats, bacteria often occur in mixed-species communities instead of mono-species cultures. One example is the co-occurrence of *P. aeruginosa* and *S. epidermidis* in patients with chronic wounds and venous ulcers^28^,^29^,^30^. Mixed-species biofilms often promote each other’s survival and show a different response to antimicrobial treatment. To investigate whether CAP can eradicate mixed-species biofilms in a similar fashion to single species, co-cultures of *P. aeruginosa* and *S. epidermidis* were treated for 1, 3 and 10 min with argon plasma using the kINPen med (Fig. 4). As for single-species biofilms, *P. aeruginosa* biofilms were more sensitive than *S. epidermidis* biofilms. However, the final CFU log_10_ reduction was only 2.6 ± 0.4 for *P. aeruginosa* when grown in co-culture with *S. epidermidis* compared to 3.6 ± 0.2 when grown as a single species. Interestingly, *S. epidermidis* had a slightly higher log_10_ reduction (1.4 ± 0.5) in mixed culture with *P. aeruginosa*, compared to 0.9 ± 0.3 log_10_ reduction in a single-species biofilm.

### CAP treatment of planktonic bacteria

**Figure 5:**
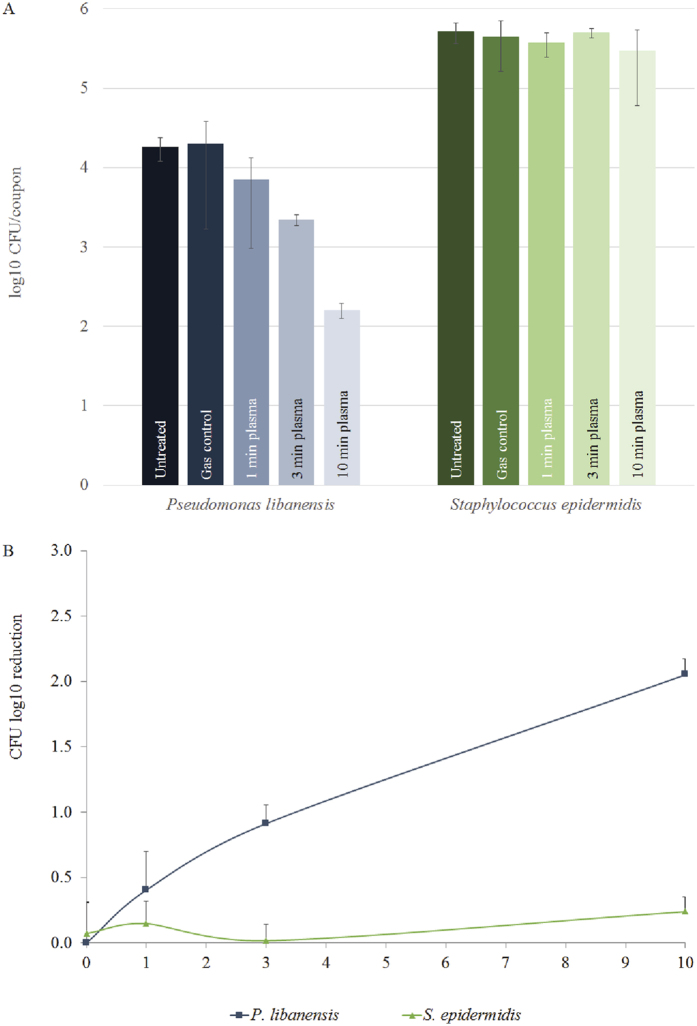
CAP treatment of planktonic cells. Overnight cultures of *P. libanensis* and *S. epidermidis* were diluted to 10^6^ CFU in fresh nutrient broth and 10 μl was placed at the centre of a sterile coupon and allowed to dry for 10 min. CAP treatment was performed for 1, 3 or 10 min using the kINPen med with argon feeding gas at 3.1 slm. After treatment, cells were scraped from the coupon and dilutions plated onto nutrient agar. (**A**) CFU numbers of untreated, gas control, 1, 3 and 10 min CAP, respectively. (**B**) CFU log_10_ reductions after 0 (gas control), 1, 3 and 10 min plasma treatment. The error bars represent standard deviations for 3 samples.

To evaluate whether species-dependent inactivation occurs only during biofilm mode of growth, the Gram positive *S. epidermidis* and the Gram negative *P. libanensis* were tested for inactivation during planktonic growth mode. A similar response compared to biofilm treatment was observed. *P. libanensis* had a significantly higher CFU log_10_ reduction after ten minute argon plasma treatment compared to *S. epidermidis* (Fig. 5).

### Optical emission spectra of kINPen med operated with argon

Emission spectra were measured to examine any qualitative changes in plasma characteristics during the treatment time.

**Figure 6:**
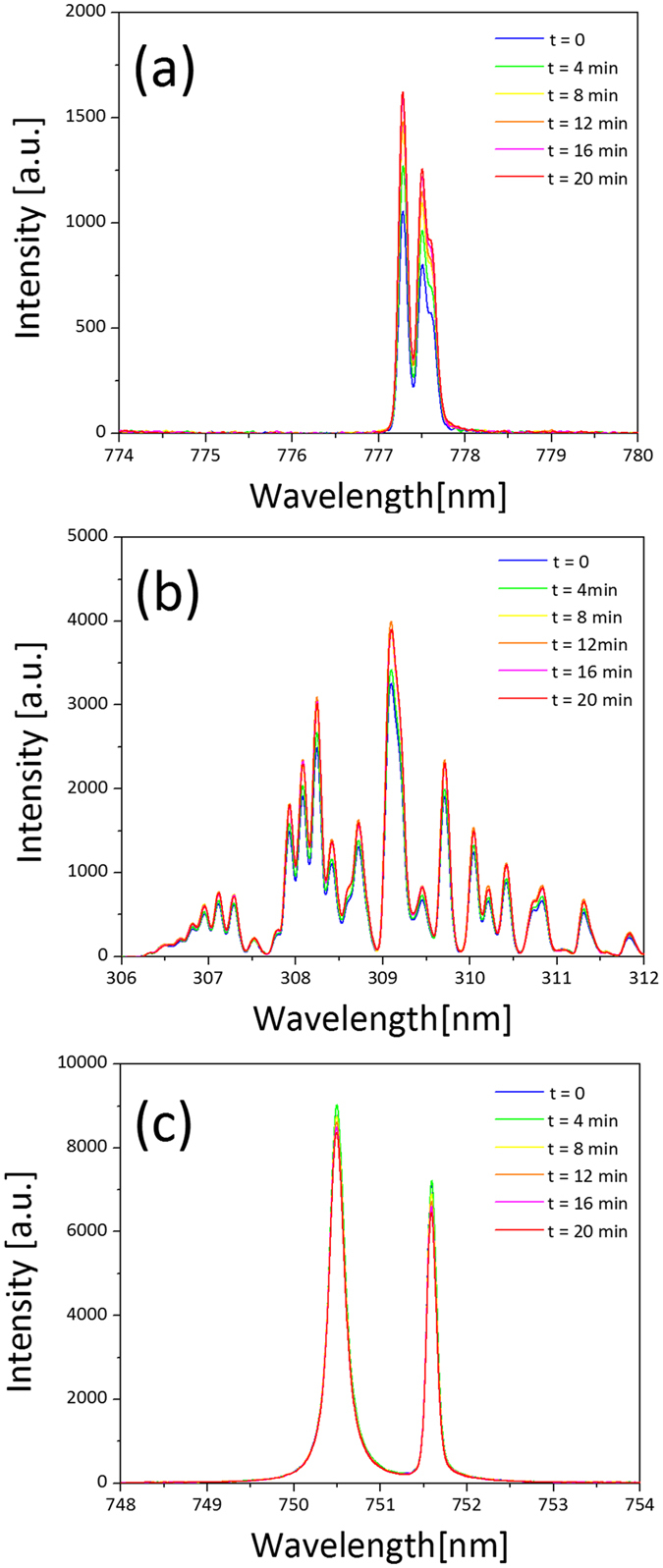
Optical emission spectra for kINPen med plasma device over 20 minutes showing atomic oxygen lines (**a**), hydroxyl radical bands (**b**) and atomic argon lines (**c**).

The presence of active OH, O and Ar radicals were identified from the spectra, as shown in Fig. 6. The typical molecular nitrogen emission from the N_2_ (C^3^Π_u_ → B^3^Π_g_) transition was very weak in comparison to OH emission. NH radical and N_2_^+^ ion emissions were not observed within the detection limit for the 10 ms exposure time setting. During the 20 min duration of the plasma discharge, the emission intensity of atomic oxygen (O I ^5^P → ^5^S) (Fig. 6a) and hydroxyl radicals (OH (A^2^Σ^+^ → X^2^Π)) (Fig. 6b) increased by approximately 15%. A slight decrease of intensity of metastable Ar emission was observed (Fig. 6c).

## Discussion

We have observed a marked difference in sensitivity to CAP treatment between Gram positive and Gram negative bacterial biofilms. The visible biofilm biomass appears similar among the six different species, with all showing a layer of single cells and small microcolonies before treatment (Fig. 1). The similar thickness and structure of the biofilms suggests that factors other than biofilm architecture are responsible for the observed variation in CAP sensitivity. The major difference between the two groups of bacteria is the thickness of the cell wall and the presence of an outer membrane in Gram negative bacteria only. The bacterial cell wall ranges from 20–80 nm thick for Gram positive and between 1.5–10 nm thick for Gram negative bacteria. The main component of the cell wall is peptidoglycan, which is found in almost all bacteria and is responsible for preserving the integrity of the cell. Destruction of peptidoglycan either through mutations or external stresses (e.g. antibiotics) will lead to cell lysis^25^,^31^.

Of the six bacteria tested, the Gram positives had a significantly higher resistance after 10 min CAP treatment (p = 0.034), with around 1 log_10_ reduction for *B. subtilis* and *S. epidermidis* and up to 2 log_10_ reduction for *K. carniphila*, suggesting that the cell wall thickness plays a role for plasma mediated inactivation time. The organism with the thickest cell wall, *B. subtilis* had the lowest log_10_ reduction after 10 min CAP treatment. For CAP treatment times of 1 and 3 minutes, the same inactivation trend of Gram positive bacteria having lower inactivation rates than Gram negative bacteria was observed. However, a correlation within the Gram groups could not be seen. For example, the Gram negative *E. cloacae* has a thicker cell wall than the Gram negative *P. aeruginosa,* but *E. cloacae* showed a higher log_10_ reduction than *P. aeruginosa* at 1 and 3 min treatment, suggesting that other factors, including the outer membrane in Gram negative and cell appendices (e.g. glycan strands) in Gram positive bacteria are likely to play a role in CAP inactivation of bacteria.

The bacterial cytoplasmic membrane consists of phospholipids and the Gram negative outer membrane consists of phospholipids and lipopolysaccharides^32^. Peroxidation of lipids is a well-known mechanism of CAP inactivation^4^,^33^. Membrane lipids have been suggested to be the macromolecules of the cell that are most vulnerable to physical stresses due to their position at the outside of the cell envelope and their sensitivity to ROS^33^. In addition, due to the presence of pore-forming proteins (porins), the outer membrane is leakier than the cytoplasmic membrane and the cell wall and thus potentially easier to penetrate by CAP, possibly leading to a higher sensitivity of Gram negative bacteria to plasma treatment.

In agreement with our observation of higher sensitivity of Gram negative cells to CAP, Laroussi *et al*. observed gross morphological changes only in Gram negative *Escherichia coli* and not in Gram positive *B. subtilis* using scanning electron microscopy after CAP treatment^34^. The study suggested that the observed cell lysis of *E. coli* is due to an electrostatic disruption of the outer cell membrane. Lysis may occur when the outer membrane has acquired a sufficient electrostatic charge that the outward electrostatic stress exceeds its tensile strength^34^,^35^. Moreover, a higher surface roughness or irregularity due to the presence of an outer membrane could render Gram negative cells more sensitive to electrostatic disruption^35^.

A recent study by Flynn *et al* tested a range of antibacterial resistant strains (ESKAPE pathogens) for sensitivity to CAP^36^. ESKAPE pathogens are *Enterococcus faecium* (Gram positive), *Staphylococcus aureus* (Gram positive), *Klebsiella pneumoniae* (Gram negative), *Acinetobacter baumannii* (Gram negative), *Pseudomonas aeruginosa* PA14 (Gram negative) and *Enterobacter cloacae* (Gram negative). As in our study, it was observed that Gram negative organisms were killed more rapidly than Gram positives for biofilm and planktonic cultures, with the exception of *Acinetobacter baumannii* (Gram negative) biofilms, which were highly resistant despite a thin (26 nm) cell wall^36^. This suggests that additional factors to cell wall thickness play a role in CAP sensitivity.

A study by Montie *et al*. has suggested that the bacterial cell wall may not be compromised by ROS from plasma, although it allows ROS to penetrate by diffusion, and only the cell membranes (cytoplasmic membrane and outer membrane) are vulnerable to physical disruption by plasma^33^. However, the diffusion across a thick Gram positive cell wall would presumably still be slower than across a thin Gram negative cell wall, leading to a difference in CAP sensitivity. In contrast, a study by Mozetic *et al*. has clearly shown damage by CAP treatment to the cell wall of *Bacillus stearothermophilus* before the plasma membrane was damaged^37^ and further indications that the bacterial cell wall is an important target during plasma interaction have been reported^13^,^14^.

Interestingly, by being operated in an open-air environment, the emission intensity of atomic oxygen and hydroxyl radicals increased without external supply of oxygen. In contrast, a slight decrease in intensity of metastable Ar emission was observed. Argon was used as the feeding gas for the plasma equipment. Oxygen and water vapour can originate from the surrounding air or from the sample being treated. The increase in atomic oxygen and hydroxyl radical density may be due to evaporation from the samples being treated. The decrease in emission intensity is likely a consequence of the efficient quenching of argon metastables by oxygen molecules and atoms^38^.

Yusupov *et al*. used reactive molecular dynamics simulations to show interactions of plasma species with peptidoglycan. It was shown that plasma species can break structurally-important bonds of peptidoglycan, ultimately leading to cell death^13^. Specifically, it was demonstrated that the presence of OH and O lead to C-O, C-N and C-C bond breakage. We have detected OH and O in the emission spectra of the kINPen for our experimental conditions (Fig. 6) making them a likely cause for peptidoglycan bond breakage in our experiments.

In some bacteria the cell wall has additional structural elements that could also influence CAP sensitivity. For example, *B. subtilis* was found to have glycan strands up to 5 μm, longer than the cell itself, and a cabling architecture^23^, suggesting a protective role during CAP treatment in addition to having a very thick cell wall.

In addition to the structural envelope of single cells, the extracellular matrix (ECM) in which biofilm cells are embedded is likely to affect CAP sensitivity. The ECM gives cells added protection to external stress. The ECM composition varies between species, but it consists mainly of extracellular polymeric substance, including polysaccharides, lipids, proteins and nucleic acids^39^. It has been suggested that the ECM composition plays an important role in susceptibility to reactive species, such as found in CAP^40^,^41^. Despite a negative charge of the ECM, which prevents many reactive reagents from penetrating, some small plasma-generated molecules were shown to successfully reach the interior of the biofilm and lead to cell death^41^. However, because of the species-specific composition of the ECM further investigations are required to assess its specific role in CAP treatment of different bacterial species.

Mixed species biofilms are often found to have increased resistance to antibiotics and disinfection agents^42^,^43^,^44^. To examine whether a similar higher resistance occurs during CAP treatment, we investigated the effect of CAP treatment on clinically-significant mixed-species biofilms of *P. aeruginosa* and *S. epidermidis. P. aeruginosa* and *S. epidermidis* are known to occur simultaneously in patients with chronic wounds and venous ulcers^28^,^29^,^30^. Our results show that similar to single-species biofilms, the Gram negative *P. aeruginosa* with a thin cell wall had a significantly higher susceptibility to CAP after 10 min of treatment compared to the Gram positive *S. epidermidis* with a thick cell wall. However, the overall reduction in CFU was lower (2.6 ± 0.4 log_10_) when grown as a mixed biofilm, compared to 3.6 ± 0.2 log_10_ when grown alone (Figs 2 and 4), suggesting a higher resistance of *P. aeruginosa* biofilms to CAP treatment in a mixed culture (Fig. 4). A recent study by Jahid *et al*.^45^ showed a similarly increased resistance of mixed species biofilms to CAP of *Salmonella typhimurium* when grown in the presence of indigenous lettuce microorganisms. Interestingly, *S. epidermidis* was more susceptible to plasma treatment when grown in a mixed biofilm with *P. aeruginosa*. It appears unlikely that the thickness of the cell wall would change depending on the presence of other organisms in a biofilm. Thus other factors possibly play a role in an altered resistance to CAP in mixed-species biofilms. One previously discussed hypothesis is an increase or a change in the composition of the ECM^45^. In our study a thicker or altered ECM produced by the Gram positive organism may provide better protection for the Gram negative bacteria within the biofilm community.

Our results show that CAP inactivation was less effective for *P. libanensis* and *S. epidermidis* planktonic cells than for their biofilm counterparts. This was surprising, because many studies show a higher resistance of biofilm cells to CAP than planktonic cells. However, results from Zhu *et al*.^46^ also showed a faster inactivation (1 min) for Candida biofilms compared to planktonic cells (4 min) of the same species. This has been attributed to the planktonic cells occupying a smaller area than biofilms during treatment. Similarly in our study, the 10 μl drop containing the planktonic cells (in the same CFU density as the biofilm CFU inoculum) does not spread over the entire coupon and a higher number of cells is therefore concentrated on a smaller area, thus potentially making CAP inactivation less efficient.

## Conclusions

Our study shows a correlation of CAP inactivation of bacteria and the thickness of the cell wall. We demonstrate that biofilms of Gram negative species with a thinner cell wall are inactivated more rapidly than biofilms of Gram positive bacteria with a thicker cell wall. While there is a good correlation and a significant difference in CAP sensitivity between Gram positive and Gram negative organisms at 10 min CAP treatment, a significant difference between the two groups of bacteria was not observed for CFU log_10_ reduction at 1 and 3 min CAP treatment. This suggests that other targets in the cell and biofilm community play a role in CAP inactivation, including ECM, cell membrane, DNA and proteins.

We further show that multi-species biofilms have a different inactivation profile compared to mono-species biofilms. Similar findings have been reported for other inactivation methods, such as antibiotics^43^,^47^. However, more evidence, including multi-species biofilms of other species, is needed to fully investigate and explain this phenomenon.

In summary, our results provide insights into the role of the bacterial cell wall during the interaction with reactive species produced by CAP. These findings have implications for the use of CAP in medical, industrial and other sterilization settings.

## Methods

### Bacterial species and culture media

A range of environmental and pathogenic bacterial strains were used in this study (Table 1). All strains were routinely cultivated on nutrient agar (1 g l^−1^ ‘Lab-Lemco’ powder, 2 g l^−1^ yeast extract, 5 g l^−1^ peptone, 5 g l^−1^ sodium chloride, 15 g l^−1^ agar, pH 7.4; Oxoid) using standard methods. Overnight cultures were inoculated into 10 ml nutrient broth (1 g l^−1^ ‘Lab-Lemco’ powder, 2 g l^−1^ yeast extract, 5 g l^−1^ peptone, 5 g l^−1^ sodium chloride, pH 7.4; Oxoid) and incubated at 150 rpm shaking at 37 °C (*Pseudomonas aeruginosa, Enterobacter cloaceae* and *Staphylococcus epidermidis*) or 30 °C (*Pseudomonas libanensis, Kocuria carniphila* and *Bacillus subtilis*), with the temperature chosen in accordance with the optimal growth conditions for the organism.

### Biofilm formation

Biofilms were allowed to form on smooth, stainless steel coupons (RD 128–316, 1.27 cm diameter, 0.3 cm thick; Biosurface Technologies, Bozeman, MT, USA) placed in 24-well microtiter plates. Overnight cultures of the respective species were grown in 10 ml nutrient broth with 150 rpm shaking. Cultures were diluted to 10^6^ CFU in fresh nutrient broth before inoculating 1 ml in 24 well plates with a stainless steel coupon inside. Each strain was inoculated in triplicates. For mixed species biofilms of *S. epidermidis* and *P. aeruginosa*, cultures were mixed to a 1:1 ratio immediately prior to biofilm inoculation.

After 24 hours incubation, coupons were washed with 1 ml PBS to remove unattached cells. Coupons were then placed in an empty petri dishes and air-dried for 10 min before plasma treatment. For *B. subtilis*, the possible presence of endospores was determined by comparing CFU numbers of a heat killed sample (spores) with a regular sample (vegetative cells and spores). It was found that only 0.01% of the total cell number was in endospore form (data not shown).

### Biofilm imaging

To visualize the biofilms, cells were stained by covering the coupon with 100 μl of the BacLight Live/dead stain containing the 2 stock solutions green Syto 9 and red propidium iodide (Molecular Probes, Eugene, Oregon, USA) according to the manufacturer’s instructions. Cells were visualised using an Olympus IX83 microscope equipped with CellSense imaging software.

### Plasma treatment and determination of CFU

Plasma treatment was performed as previously described^10^. Briefly, plasma treatment was conducted using the kINPen med (Neoplas tools GmbH, Greifswald, Germany) with argon as the feed gas at 3.1 slm^7^,^11^. Plasma was applied to the centre of the coupon, with a distance of 1 cm between the tip of the kINPen device and the coupon. The coupon was not moved during treatment. As a control, a 10 min gas treatment without igniting plasma was performed. Samples were treated in triplicates for 1, 3 and 10 min, respectively.

After treatment, coupons were placed into 3 ml sterile phosphate-buffered saline (PBS). The coupon surface was scraped and vigorously mixed with a sterile spatula to remove cells. In addition, coupons were placed in an ultrasonic bath (Vibron USB08CD; Galsonic Pty. Ltd., SA, Australia) for 3 min to dissolve possible cell clumps. It was confirmed that sonication did not affect cell viability (data not shown). Two dilution series were made for each treated biofilm sample. CFU counts were determined in triplicate by the drop plate method as previously described^48^. Following serial dilution, cells were plated onto nutrient agar and incubated for 24 h at 37 °C or 30 °C, followed by another 2 days at room temperature before counting CFU. For *S. epidermidis* and *P. aeruginosa* mixed cultures, dilutions were plated onto selective Pseudomonas (Oxoid) and Mannitol salt agar (Oxoid) to distinguish the strains. All experiments were performed at least twice in triplicate, with similar results. Data presented in the manuscript are those from one set of experiments with error bars corresponding to the standard deviation from 3 independently-grown biofilms, 2 dilution series from each biofilm and 3 CFU droplets.

Log_10_ reduction values were calculated by subtracting Log_10_ (CFU/coupon of plasma treatment time) from Log_10_ (CFU/coupon of control).

A two-tailed, two sample (unequal variance) Student’s t-test with a significance level 0.05 was performed comparing log10 reduction values of the three Gram positive strains to log10 reduction values of the three Gram negative strains for each of the plasma treatment times.

### Planktonic cell growth and plasma treatment

For treatment of planktonic cells, cells were grown overnight in 10 ml nutrient broth in falcon tubes with shaking at 150 rpm at either 37 °C or 30 °C, respectively. Immediately prior to plasma treatment, cultures were diluted to 10^6^ CFU in fresh nutrient broth and 10 μl was placed at the centre of a sterile coupon and allowed to dry for 10 min. Two dilution series were made for each treated sample. CFU counts were determined in triplicates by the drop plate method as previously described^48^. All experiments were performed at least twice in triplicates with similar results. Data presented are that of one round of experiments with error bars corresponding to 3 independently grown cultures, 2 dilution series from each culture and 3 CFU droplets.

### Optical emission spectra

In order to investigate any changes in plasma characteristics during treatment time, light emission spectra were measured using an optical emission spectrometer (Princeton Instruments Acton SP2500) equipped with 1200 grooves/mm triple gratings to enable measurements from 190 nm to more than 1 μm wide wavelength range in a typical setting. The spectra were measured with the entrance slit 10 cm away from the centre of the plasma plume under similar conditions to the treatment set up. Measurements were taken in the dark at 4 min intervals over 20 min total time at room temperature with a sample underneath. The background was subtracted from the raw measurements.

## Additional Information

**How to cite this article**: Mai-Prochnow, A. *et al*. Gram positive and Gram negative bacteria differ in their sensitivity to cold plasma. *Sci. Rep.* **6**, 38610; doi: 10.1038/srep38610 (2016).

**Publisher's note:** Springer Nature remains neutral with regard to jurisdictional claims in published maps and institutional affiliations.

## Figures and Tables

**Table 1:** Bacterial species used.

Species	Gram Stain	Reference/source
*Pseudomonas aeruginosa* PAO1	Negative	49
*Pseudomonas libanensis* CIP^1^ 105460	Negative	50
*Enterobacter cloaceae* ATCC^2^ 13047 T	Negative	50
*Kocuria carniphila* CCM^3^ 132	Positive	50
*Staphylococcus epidermidis* FRRB^4^ 2505	Positive	FRRB^4^
*Bacillus subtilis* ATCC^2^ 6051	Positive	FRRB^4^

^1^Collection of Institut Pasteur.

^2^American Type Culture Collection.

^3^Czech Collection of Microorganisms.

^4^Food Research Ryde Bacteriology (CSIRO Food and Nutrition) culture collection.

## References

[b1] Graves D. B. The emerging role of reactive oxygen and nitrogen species in redox biology and some implications for plasma applications to medicine and biology. Journal of Physics D: Applied Physics 45, 263001 (2012).

[b2] Moisan M. . Plasma sterilization. Methods mechanisms. Pure and Applied Chemistry 74, 349–358 (2002).

[b3] Oehmigen K. . The Role of Acidification for Antimicrobial Activity of Atmospheric Pressure Plasma in Liquids. Plasma Process Polym 7, 250–257 (2010).

[b4] Vatansever F. . Antimicrobial strategies centered around reactive oxygen species - bactericidal antibiotics, photodynamic therapy, and beyond. FEMS microbiology reviews 37, 955–989 (2013). 23802986 10.1111/1574-6976.12026 PMC3791156

[b5] von Woedtke T., Metelmann H. R. & Weltmann K. D. Clinical Plasma Medicine: State and Perspectives of *in vivo* Application of Cold Atmospheric Plasma. Contributions to Plasma Physics 54, 104–117 (2014).

[b6] Isbary G. . Non-thermal plasma—More than five years of clinical experience. Clinical Plasma Medicine 1, 19–23 (2013).

[b7] Winter J., Brandenburg R. & Weltmann K. D. Atmospheric pressure plasma jets: an overview of devices and new directions. Plasma Sources Science and Technology 24, 064001 (2015).

[b8] Weltmann K. D., Kindel E., Brandenburg R. & Meyer C. Atmospheric Pressure Plasma Jet for Medical Therapy: Plasma Parameters and Risk Estimation. Contributions to plasma physics 49, 631–640 (2009).

[b9] Metelmann H.-R. . Scar formation of laser skin lesions after cold atmospheric pressure plasma (CAP) treatment: A clinical long term observation. Clinical Plasma Medicine 1, 30–35 (2013).

[b10] Mai-Prochnow A., Bradbury M., Ostrikov K. & Murphy A. B. *Pseudomonas aeruginosa* Biofilm Response and Resistance to Cold Atmospheric Pressure Plasma Is Linked to the Redox-Active Molecule Phenazine. Plos One 10, e0130373 (2015). 26114428 10.1371/journal.pone.0130373 PMC4483161

[b11] Bussiahn R., Woedtke T.v. & Weltmann K. D. In Plasma Science (ICOPS), 2012 Abstracts IEEE International Conference on 7E-3-7E-3 (2012).

[b12] Mai-Prochnow A., Murphy A. B., McLean K. M., Kong M. G. & Ostrikov K. Atmospheric pressure plasmas: Infection control and bacterial responses. International journal of antimicrobial agents 43, 508–517 (2014). 24637224 10.1016/j.ijantimicag.2014.01.025

[b13] Yusupov M. . Plasma-Induced Destruction of Bacterial Cell Wall Components: A Reactive Molecular Dynamics Simulation. The Journal of Physical Chemistry C 117, 5993–5998 (2013).

[b14] Yusupov M. . Atomic-scale simulations of reactive oxygen plasma species interacting with bacterial cell walls. New Journal of Physics 14, 093043 (2012).

[b15] Joshi S. G. . Nonthermal dielectric-barrier discharge plasma-induced inactivation involves oxidative DNA damage and membrane lipid peroxidation in *Escherichia coli*. Antimicrobial agents and chemotherapy 55, 1053–1062 (2011). 21199923 10.1128/AAC.01002-10 PMC3067084

[b16] Boles B. R., Thoendel M. & Singh P. K. Self-generated diversity produces “insurance effects” in biofilm communities. Proc Natl Acad Sci USA 101, 16630–16635 (2004). 15546998 10.1073/pnas.0407460101 PMC528905

[b17] Branda S. S., Vik S., Friedman L. & Kolter R. Biofilms: the matrix revisited. Trends in microbiology 13, 20–26 (2005). 15639628 10.1016/j.tim.2004.11.006

[b18] Burmolle M. . Biofilms in chronic infections - a matter of opportunity - monospecies biofilms in multispecies infections. FEMS immunology and medical microbiology 59, 324–336 (2010). 20602635 10.1111/j.1574-695X.2010.00714.x

[b19] Conibear T. C., Collins S. L. & Webb J. S. Role of mutation in *Pseudomonas aeruginosa* biofilm development. Plos One 4, e6289 (2009). 19606212 10.1371/journal.pone.0006289 PMC2705801

[b20] Costerton J. W., Stewart P. S. & Greenberg E. P. Bacterial biofilms: a common cause of persistent infections. Science (New York, NY) 284, 1318–1322 (1999). 10.1126/science.284.5418.1318 10334980

[b21] Matias V. R. F., Al-Amoudi A., Dubochet J. & Beveridge T. J. Cryo-Transmission Electron Microscopy of Frozen-Hydrated Sections of *Escherichia coli* and *Pseudomonas aeruginosa*. J Bacteriol 185, 6112–6118 (2003). 14526023 10.1128/JB.185.20.6112-6118.2003 PMC225031

[b22] Nunes A. P. F. . Heterogeneous resistance to vancomycin in *Staphylococcus epidermidis, Staphylococcus haemolyticus* and *Staphylococcus warneri* clinical strains: characterisation of glycopeptide susceptibility profiles and cell wall thickening. International journal of antimicrobial agents 27, 307–315 (2006). 16542825 10.1016/j.ijantimicag.2005.11.013

[b23] Hayhurst E. J., Kailas L., Hobbs J. K. & Foster S. J. Cell wall peptidoglycan architecture In Bacillus subtilis. Proceedings of the National Academy of Sciences 105, 14603–14608 (2008). 10.1073/pnas.0804138105 PMC2567149 18784364

[b24] Matias V. R. & Beveridge T. J. Cryo-electron microscopy reveals native polymeric cell wall structure in *Bacillus subtilis* 168 and the existence of a periplasmic space. Mol Microbiol 56, 240–251 (2005). 15773993 10.1111/j.1365-2958.2005.04535.x

[b25] Vollmer W., Blanot D. & De Pedro M. A. Peptidoglycan structure and architecture. FEMS microbiology reviews 32, 149–167 (2008). 18194336 10.1111/j.1574-6976.2007.00094.x

[b26] Eumkeb G. & Chukrathok S. Synergistic activity and mechanism of action of ceftazidime and apigenin combination against ceftazidime-resistant *Enterobacter cloacae*. Phytomedicine 20, 262–269 (2013). 23218402 10.1016/j.phymed.2012.10.008

[b27] Coward J. E. & Rosendranz H. S. Electron microscopic appearance of silver sulfadiazine-treated *Enterobacter cloacae*. Chemotherapy 21, 231–235 (1975). 1157574 10.1159/000221863

[b28] Gjødsbøl K. . Multiple bacterial species reside in chronic wounds: a longitudinal study. International Wound Journal 3, 225–231 (2006). 16984578 10.1111/j.1742-481X.2006.00159.x PMC7951738

[b29] Marra A. R., Bearman G. M. L., Wenzel R. P. & Edmond M. B. Comparison of the systemic inflammatory response syndrome between monomicrobial and polymicrobial *Pseudomonas aeruginosa* nosocomial bloodstream infections. Bmc Infect Dis 5 (2005). 10.1186/1471-2334-5-94 PMC1289283 16259623

[b30] Pihl M., Chávez de Paz L. E., Schmidtchen A., Svensäter G. & Davies J. R. Effects of clinical isolates of *Pseudomonas aeruginosa* on *Staphylococcus epidermidis* biofilm formation. FEMS Immunology & Medical Microbiology 59, 504–512 (2010). 20579097 10.1111/j.1574-695X.2010.00707.x

[b31] Rogers H., Perkins H. R. & Ward J. B. Microbial Cell Walls and Membranes. (Springer Netherlands, Dordrecht; 1980).

[b32] Koebnik R., Locher K. P. & Van Gelder P. Structure and function of bacterial outer membrane proteins: barrels in a nutshell. Mol Microbiol 37, 239–253 (2000). 10931321 10.1046/j.1365-2958.2000.01983.x

[b33] Montie T. C., Kelly-Wintenberg K. & Roth J. R. An overview of research using the one atmosphere uniform glow discharge plasma (OAUGDP) for sterilization of surfaces and materials. Ieee T Plasma Sci 28, 41–50 (2000).

[b34] Laroussi M., Mendis D. A. & Rosenberg M. Plasma interaction with microbes. New Journal of Physics 5, 41 (2003).

[b35] Mendis D. A., Rosenberg M. & Azam F. A note on the possible electrostatic disruption of bacteria. Plasma Science, IEEE Transactions on 28, 1304–1306 (2000).

[b36] Flynn P. B. . Bactericidal efficacy of atmospheric pressure non-thermal plasma (APNTP) against the ESKAPE pathogens. International journal of antimicrobial agents 46, 101–107 (2015). 25963338 10.1016/j.ijantimicag.2015.02.026

[b37] Mozetic M. & Vratnica Z. Destruction of *Bacillus stearothermophilus* bacteria by weakly ionized low pressure cold oxygen plasma. Vacuum 85, 1080–1082 (2011).

[b38] Rauf S. & Kushner M. J. Argon metastable densities in radio frequency Ar, Ar/O-2 and Ar/CF4 electrical discharges. J Appl Phys 82, 2805–2813 (1997).

[b39] Flemming H. C. & Wingender J. The biofilm matrix. Nat Rev Microbiol 8, 623–633 (2010). 20676145 10.1038/nrmicro2415

[b40] Delben J. A., Zago C. E., Tyhovych N., Duarte S. & Vergani C. E. Effect of Atmospheric-Pressure Cold Plasma on Pathogenic Oral Biofilms and *in vitro* Reconstituted Oral Epithelium. Plos One 11, e0155427 (2016). 27224027 10.1371/journal.pone.0155427 PMC4880209

[b41] Traba C. & Liang J. F. Susceptibility of *Staphylococcus aureus* biofilms to reactive discharge gases. Biofouling 27, 763–772 (2011). 21774615 10.1080/08927014.2011.602188 PMC3181119

[b42] van der Veen S. & Abee T. Mixed species biofilms of *Listeria monocytogenes* and *Lactobacillus plantarum* show enhanced resistance to benzalkonium chloride and peracetic acid. International Journal of Food Microbiology 144, 421–431 (2011). 21084128 10.1016/j.ijfoodmicro.2010.10.029

[b43] Lopes S. P., Ceri H., Azevedo N. F. & Pereira M. O. Antibiotic resistance of mixed biofilms in cystic fibrosis: impact of emerging microorganisms on treatment of infection. International journal of antimicrobial agents 40, 260–263 (2012). 22770521 10.1016/j.ijantimicag.2012.04.020

[b44] Zameer F. & Gopal S. Evaluation of Antibiotic Susceptibility in Mixed Culture Biofilms. International Journal of Biotechnology and Biochemistry 6, 93–99 (2010).

[b45] Jahid I. K., Han N., Zhang C.-Y. & Ha S.-D. Mixed culture biofilms of *Salmonella Typhimurium* and cultivable indigenous microorganisms on lettuce show enhanced resistance of their sessile cells to cold oxygen plasma. Food Microbiology 46, 383–394 (2015). 25475308 10.1016/j.fm.2014.08.003

[b46] Zhu W.-D. . In Plasma for Bio-Decontamination, Medicine and Food Security. (eds. Machala Z., Hensel K. & Akishev Y.) 201–214 (Springer Netherlands, Dordrecht; 2012).

[b47] Narisawa N., Haruta S., Arai H., Ishii M. & Igarashi Y. Coexistence of Antibiotic-Producing and Antibiotic-Sensitive Bacteria in Biofilms Is Mediated by Resistant Bacteria. Appl Environ Microb 74, 3887–3894 (2008). 10.1128/AEM.02497-07 PMC2446560 18441106

[b48] Chen C. Y., Nace G. W. & Irwin P. L. A 6 × 6 drop plate method for simultaneous colony counting and MPN enumeration of *Campylobacter jejuni, Listeria monocytogenes*, and *Escherichia coli*. Journal of Microbiological Methods 55, 475–479 (2003). 14529971 10.1016/s0167-7012(03)00194-5

[b49] Jacobs M. A. . Comprehensive transposon mutant library of *Pseudomonas aeruginosa*. Proc Natl Acad Sci 100, 14339–14344 (2003). 14617778 10.1073/pnas.2036282100 PMC283593

[b50] Knight G. C. & Craven H. M. A model system for evaluating surface disinfection in dairy factory environments. Int J Food Microbiol 137, 161–167 (2010). 20022125 10.1016/j.ijfoodmicro.2009.11.028

